# When Age Matters: Differences in Facial Mimicry and Autonomic Responses to Peers' Emotions in Teenagers and Adults

**DOI:** 10.1371/journal.pone.0110763

**Published:** 2014-10-22

**Authors:** Martina Ardizzi, Mariateresa Sestito, Francesca Martini, Maria Alessandra Umiltà, Roberto Ravera, Vittorio Gallese

**Affiliations:** 1 Department of Neuroscience, Unit of Physiology, University of Parma, Parma, Italy; 2 Department of Psychology - Clinical Psychology Unit, ASL1 Imperiese, Imperia, Italy; Harvard Medical School, United States of America

## Abstract

Age-group membership effects on explicit emotional facial expressions recognition have been widely demonstrated. In this study we investigated whether Age-group membership could also affect implicit physiological responses, as facial mimicry and autonomic regulation, to observation of emotional facial expressions. To this aim, facial Electromyography (EMG) and Respiratory Sinus Arrhythmia (RSA) were recorded from teenager and adult participants during the observation of facial expressions performed by teenager and adult models. Results highlighted that teenagers exhibited greater facial EMG responses to peers' facial expressions, whereas adults showed higher RSA-responses to adult facial expressions. The different physiological modalities through which young and adults respond to peers' emotional expressions are likely to reflect two different ways to engage in social interactions with coetaneous. Findings confirmed that age is an important and powerful social feature that modulates interpersonal interactions by influencing low-level physiological responses.

## Introduction

An accurate recognition and classification of emotional facial expressions is highly relevant for humans and their social interactions. Previous studies demonstrated that explicit recognition of human facial expressions is influenced by group membership [1], [2]. In this regard, ethnic group membership [3]–[6] and gender [7]–[10] are two studied factors which modulate face decoding and emotional facial expressions recognition. Another relevant feature of human faces that determines group membership is age. In all societies age is a relevant characteristic, contributing to status ascription and markedly influencing social interactions. Interestingly, it has been demonstrated that Age-group membership affects explicit face decoding and emotional facial expressions recognition [11]. Indeed, faces belonging to someone being the same age of the perceiver are better remembered [12], capture greater attention [13] and induce longer looking time, resulting in a better emotional expressions identification [14]. Overall, these findings suggest that individuals are more likely to attend and to explicitly respond to social signals coming from peers, than to those coming from older or younger individuals. An untested hypothesis is whether Age-group membership could also affect the implicit physiological responses to emotional facial expressions. A possible modulation of physiological responses consequent to an of Age-group membership effect would indicate that age operates also at a pre-reflective, automatic and unconscious level, thus opening new intriguing avenues, in the investigation of the evolutionarily-determined physiological responses implicated in the regulation of social behaviour.

Two automatic, low-level physiological measures are considered to be relevant for emotional facial perception: facial mimicry and autonomic regulation. Negative and positive emotional facial expressions induce in the observer an automatic, unconscious and rapid facial electromyographic (EMG) response in the same muscles involved in expressing the observed emotion [15]. This phenomenon, called “facial mimicry” has been proposed to facilitate empathy, emotional reciprocity and recognition, thus characterizing interpersonal relationships in a meaningful, affective fashion [16]. In other words, facial mimicry serves to automatically and non-consciously synchronize people's emotional disposition and promote social cohesion, however it can also be motivationally driven. The nature of the social contexts, such as Group membership, has been demonstrated that could modulate automatic facial mimicry. Indeed, some studies, investigating the influence of social affiliation group membership on facial mimicry, reported greater facial mimicry in response to negative facial expressions displayed by in-group members with respect to those expressed by out-group members [17]–[19]. Nevertheless, no study investigated whether, besides cultural and social affiliations like political, professional and educative membership, also biological features of human beings, such as age, which implicitly influences social relationships, could induce a specific Age-group membership effect on facial mimicry.

During social interactions, others' facial expressions not only provoke an automatic facial mimicry response in the observer, but also contribute to define the nature of the situation in which people are engaged. In other words, other people's facial expressions allow the observer to understand whether the contingent social environment is dangerous or safe, that is, whether threatening stimuli has to be expected or not. Hence, facial expressions are essential information in order to implement consistent behavioral adaptations to the external environment. Behavioral regulation requires a synchronous and overall control of the entire body, which is carry out by the Autonomic Nervous System. For this reason, the evaluation of human autonomic regulation during social interactions, in addition to the recording of facial mimicry, could disclose relevant information about the regulation of social behavior. The parasympathetic and sympathetic autonomic nervous subsystems represent antagonist, but coordinated, regulation mechanisms by which an appropriate internal state meets shifts in both internal and external demands. The parasympathetic subsystem promotes a calm state consistent both with metabolic demands of growth and restoration and with social interactions. The main actor of the parasympathetic subsystem is the Vagus Nerve. The myelinated branch of the Vagus Nerve, which humans share with some mammals living in herds, thanks to the control of face striated muscles and of several visceral organs, contributes to the richness of human social behaviour. For example the myelinated branch of the Vagus Nerve is implicated in low face expressivity, eye contact, prosody expression and middle ear muscles modulation to improve the extraction of human voice [20]. Respiratory Sinus Arrhythmia (RSA) is one of the periodic components of heart rate variability resulting from the coupling of cardiovascular and respiratory systems by which the ECG R-R intervals are shortened during inspiration and prolonged during expiration [21]. It is formally defined as the heart rate variance (measured as R-R interval expressed in msec) across the band of frequencies associated with spontaneous respiration (0.12–0.40 Hz) [22]. This modulation of heart rate in synchrony with respiration is physiologically carried out by the myelinated branch of the Vagus Nerve. Hence, RSA is defined as a valid index of the vagal influence on the heart [20]. Being this branch particularly implicated in the autonomic regulation of numerous social behaviours as previously described, RSA is considered an indirect but consistent measure of humans' ability to adapt their autonomic responses to the environmental social stimuli and to establish a physiological state suitable for social relations (i.e., “self-regulation” and “social disposition” skills) [23]. From this perspective, the RSA recording, rather than other cardiac parameters like Heart Rate Variability, Toichi index [24] or Cardiac Coherence [25], allows the measurement of specific aspects of the autonomic regulation primarily involved in social behaviors. Coherently, individuals with low RSA and/or poor RSA regulation exhibit difficulties in regulating emotional state, in appropriately attending to social cues and gestures, and in expressing contingent and appropriate emotions [26]. A significant RSA modulation has also been recorded during social interactions in function of social distance [27]. Up to now, however, nobody ever explored if group membership could also influence autonomic regulation when individuals perceive either in-group or out-group members' emotional facial expressions.

The aim of this study is to test a novel hypothesis investigating whether facial mimicry and autonomic regulation to emotional facial expressions are affected by Age-group membership. To this purpose, teenager and adult participants viewed five facial expressions (anger, fear, joy, sadness and neutral) performed by both teenager and adult models while facial EMG and RSA responses were measured. We chose teenager and adult groups for two main reasons: the sharp distinctions between them from a developmental, social and hierarchical point of view and their engagement in relevant and frequent social interactions with their peers. We expected to find higher facial mimicry and greater autonomic regulation in response to emotional facial expressions displayed by individuals belonging to the observers' own age-group.

## Method

### Participants

Twenty teenager (Teenager-Group, TG: 10 males; 15–19 years old; mean age 16.85 years; SE 0.25) and 20 adult (Adult-Group, AG: 9 males; 45–55 years old; mean age 49.65 years; SE 0.96) participants took part to the study. Teenager participants were recruited among students of three different high schools. Adult participants were recruited among employees of ASL1 Imperiese Health Departments. The research project has been extensively illustrated in the schools and in Health Departments before all participants voluntarily accepted to be involved in the study. They did not receive any reimbursement or other types of compensation for their participation. The sample size exceeded the minimum amount required estimated by means of statistical power analysis [28] (*a priori* sample size *n* evaluated for 1-ß = 0.95, α = 0.05 and effect size  = 0.25). We suspend the sampling when we obtained two sex-balanced groups exceeding the minimum amount required 30% (i.e. common percentage of participants discarded from analysis due to artefacts). In order to control participants' health conditions and to verify exclusion criteria (i.e. cardio-respiratory or psychiatric diseases, substances abuse interfering with the cardio-respiratory activity and the habit to smoke more than 25 cigarettes per day) [27] they were asked to fill an anamnestic questionnaire. All participants had a normal or corrected to normal vision. To assure that the two groups were homogenous for cognitive and emotional features, immediately before the beginning of the study all participants were asked to fill the following questionnaires: Progressive Matrices (PM) [29], Empathy Quotient (EQ) [30], Toronto Alexithymia Scale (TAS-20) [31] and the Interpersonal Reactivity Index (IRI) [32]. All participants had IQ scores in the normal range (100±2 SD). No significant differences were found between them in EQ (t_38_ = −.748; p = 0.459), TAS-20 (t_38_ = 1.164; p = 0.252) and IRI (t_38_ = −.846; p = 0.403) scores. For participants' demographic information and questionnaires scores see Table 1. Seventy-five percent of adult participants were parents (sons/daughters mean age: 19.80 years, SE 1.27, range 6–30). All teenager participants lived with both parents and all adult participants, if parents, lived with their offspring.

**Table 1:** Participants' demographic information and questionnaires scores.

	n.	Males	Age in years	EQ	TAS-20	IRI
**AG**	20	9	49.65 (0.96)	44.10 (2.35)	54.00 (2.23)	67.85 (2.43)
**TG**	20	10	16.85 (0.25)	41.75 (2.10)	57.15 (1.53)	64.30 (3.42)

Standard errors are given in parenthesis. AG  =  Adult-Group, TG  =  Teenager-Group, EQ  =  Empathy Quotient, TAS-20  =  Toronto Alexithymia Scale, IRI  =  Interpersonal Reactivity Index.

### Stimuli

Stimuli were 60 video-morphing showing teenager (Teenager-stimuli, see Movie S1 for an illustrative Teenager-stimulus) and adult (Adult-stimuli, see Movie S2 for an illustrative Adult-stimulus) individuals, balanced for gender, performing different facial emotional expressions. The age of individuals depicted in Teenager-stimuli (15–19 years) matched the age of participants belonging to TG. Similarly, the age of individuals depicted in Adult-stimuli (45–55 years) matched the age of participants belonging to AG. The facial expressions displayed in both Teenager-stimuli and Adult-stimuli showed the transition from neutral to anger, fear, joy, sadness or another neutral facial expressions. The neutral stimuli consisted in a neutral facial expression morphed into a different neutral facial expression performed by the same model. Two different neutral expressions were used to assure dynamism also in stimuli showing unemotional facial expressions. Each emotional and neutral facial expression consisted in 12 different videos (12 different models), each one lasting 5 sec (10 fps; 1000×666 pixels), among which 6 were Teenager-stimuli (3 males) and 6 were Adult-stimuli (3 males). To make stimuli more genuine, the final 1 sec of each video-morphing consisted of the full 100% still facial expression of the same emotion or neutral expression [33]. All stimuli employed in this study were selected by means of a validation experiment previously carried out independently from the current experiment (see Text S1 for a detailed description of stimuli validation). Stimuli were presented using E-Prime 2.0 software (Psychology Software Tools, Inc).

### Procedure

**Figure 1:**
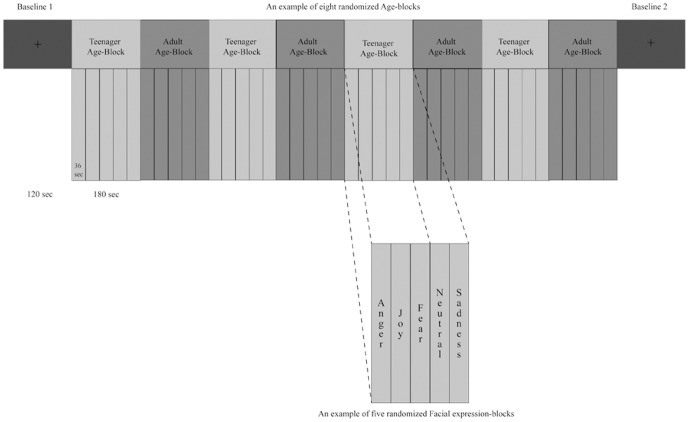
Graphical representation of the experimental procedure.

Participants were asked to abstain from alcohol, caffeine and tobacco for 2 hours prior to the experiments [27]. Participants sat comfortably in a chair in front of a laptop screen (1024×768@75 Hz) used for stimuli presentation, located at a distance of 60 cm. Participants were invited to relax and refrain from moving during the experiment. The experiment consisted of 8 “Age-blocks” (each lasting 180 sec), 4 for each age-condition (Teenager and Adult), randomly presented (see Figure 1). During Teenager Age-blocks only Teenager-stimuli were presented. On the contrary, Adult Age-blocks consisted in Adult-stimuli only. Within each Age-block, 5 “Facial expression-blocks” (one for each facial expression: 4 emotional and 1 neutral; each lasting 36 sec), were randomly presented. In each Facial expression-block six stimuli, displaying the same facial expression performed by different models (i.e., joy, fear, anger, sadness or neutral facial expressions) were shown. Each video clip was preceded by a fixation cross lasting 0.5 sec. Participants were instructed to carefully watch the videos. In order to maintain their attention, after each Age-block participants were asked a question about the physical outlook of individuals portrayed in the videos (e.g., “Did you see a woman with curly hair?”). Two “Baseline-blocks” (each lasting 120 sec) - consisting in a black centred fixation cross placed against a gray background - were presented, one at the beginning (Baseline 1) and one at the end (Baseline 2) of the experiment. During Baseline-blocks participants were asked to watch the cross. Physiological responses (EMG and ECG) were recorded for the entire duration of the experiment, that lasted about 40 min. During the experiment participants were video-recorded.

### Facial EMG recording and analysis

Facial EMG activity was bipolarly recorded on the left side of the face from the Corrugator Supercilii and the Zygomaticus Major muscle regions [34]. Before attaching the pre-gelled electrodes (4 mm standard Ag/Ag-Cl) participants' skin was cleaned with an alcohol solution. Data were converted and amplified by means of PowerLab 8/30 and Octal Bio Amp support (ADInstruments UK), and displayed, stored, and reduced with LabChart 7.3.1 software package (ADInstruments, 2011). Facial EMG was sampled at 2 kHz and recorded with an online Mains Filter. A 20–500 Hz band-pass filter [35] was applied offline to the raw facial EMG signal. EMG signals were screened for artifacts in two ways. First, a blind coder deleted trials with artifacts due to electrical noise (less than 4% of trials were deleted). Second, the video recordings of participants' faces were blindly inspected to remove trials affected by motion artifacts (i.e., a variety of facial movements not directly related to stimuli observation but affecting the EMG signal like cough, sneeze, yawn). The total average percentage of removed trials was 19.60%±10.80. The average amplitude of the EMG signal was obtained with the root-mean-square method [34]. Following standard practice [36], EMG response (expressed in microvolt, µV) was measured as change scores representing the difference between activity during each 0.5 sec of the 5 sec stimulus period and the 0.5 sec of the fixation cross immediately preceding stimulus onset.

### RSA recording and analysis

The ECG recording was performed by means of the same hardware used for Facial EMG recordings. Three 10 mm Ag/AgCl pre-gelled electrodes (ADInstruments, UK) were placed on both wrists and on the left malleolus of participants in an Einthoven's triangle configuration. The ECG was sampled at 1 KHz and online filtered with Mains Filter. Offline, the peaks of ECG R-waves were detected from each sequential heartbeat and checked by visual inspection and threshold assignment in order to identify possible artefacts. R-R intervals were extracted and eventually edited by integer division or summation [21]. The amplitude of RSA was next quantified using CMetX software (freely available from http://jallen.faculty.arizona.edu/resources_and_downloads; see [34]). RSA values [expressed in ln(msec)^2^] were calculated following this procedure: a) linear interpolation at 10 Hz sampling rate; b) application of a 241-point FIR filter with a 0.12–0.40 Hz band-pass; c) extraction of the band passed variance; d) transformation of the variance in its natural logarithm [22]. This procedure was conducted for each Facial expression-block separately (each lasting 36 sec), and for the two Baseline-blocks. To assure an homogeneous computation of RSA values, the entire duration of each Baseline-block (lasting 120 sec) was actually split in 4 consecutive epochs lasting 30 sec each [21]. In order to counterbalance the presence of RSA suppression (see Text S2 for a detailed description), RSA-response [expressed in Δ ln (msec)^2^] to each Facial expression-block was computed as the change score between RSA value of each emotional Facial expression-block (anger, fear, joy and sadness Facial expression-blocks) and the neutral Facial expression-block belonging to the same Age-block (see Text S2 for details).

For all performed analyses, p values <0.05 were considered to be statistically significant. As index of effect size we reported partial eta squared values (n^2^_p_) [37]. Post-hoc comparisons using the Newman-Keuls test were applied on significant main effects and interactions.

### Ethics Statement

All participants gave written informed consent before entering the study, which was approved by the local ethics committee of the ASL N.1 Imperiese and performed in accordance with The Code of Ethics of the World Medical Association (Declaration of Helsinki 2013) for experiments involving humans. Adult participants issued the written informed consent before participating in the study. The involvement of minors was accepted by the ethics committee, the written informed consent was obtained from underage participants as well as from their parents or guardians.

## Results

### EMG results

EMG analysis was conducted on 36 individuals (18 TG, 18 AG), because 4 participants were removed due to the high percentage of trials discarded because of artefacts. The percentage of discarded trials for excluding participants from subsequent analyses was established as more than one standard deviation above the population average (i.e., more than 30% of trials discarded). Separately for each muscle (Corrugator and Zygomaticus), two repeated measures ANOVAs were performed on facial EMG responses with Group (TG, AG) as between-factor and with Stimuli-Age (Teenager-stimuli, Adult-stimuli), Emotion (Anger, Fear, Joy, Sadness and Neutral) and Epoch (10 epochs lasting 0.5 sec) as within-factors.

**Figure 2:**
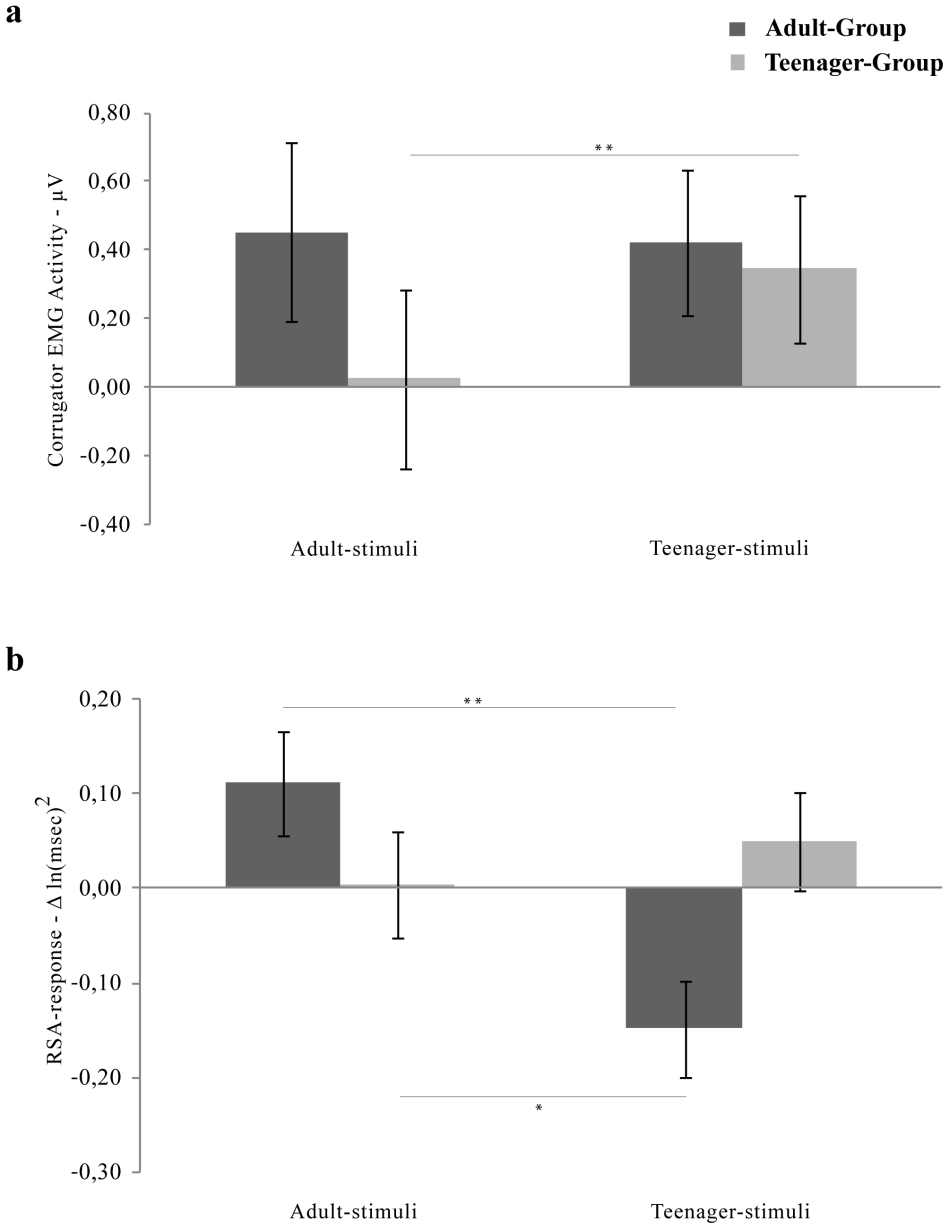
Group differences in physiological responses to peers' emotions. Panel (a) - Corrugator EMG activity expressed by Adult-Group and Teenager-Group, to Adult-stimuli and Teenager-stimuli. Panel (b) - RSA-response of Adult-Group and Teenager-Group, to Adult-stimuli and Teenager-stimuli. Error bars represent SE. * = p<0.05, ** = p<0.005.

Repeated measures ANOVA conducted on Corrugator EMG activity revealed the significant main effects of Stimuli-Age (F_1,34_ = 4.47 p = 0.041; n^2^_p_ = 0.12) and Emotion (F_4,136_ = 3.82 p = 0.005; n^2^_p_ = 0.10) factors. Furthermore, the interactions Stimuli-Age by Group (F_1,34_ = 6.56 p = 0.015; n^2^_p_ = 0.16), Stimuli-Age by Epoch (F_9,306_ = 2.11 p = 0.028; n^2^_p_ = 0.06) and Emotion by Epoch (F_36,1224_ = 2.30 p = 0.000; n^2^_p_ = 0.06) were significant. Post hoc comparisons conducted on the main effect of Stimuli-Age showed that, regardless of group membership, all participants showed higher EMG response to Teenager-stimuli (0.38 µV; SE 0.15; 95% CI 0.08 to 0.69) than to Adult-stimuli (0.24 µV; SE 0.18; 95% CI −0.14 to 0.61) (p = 0.042). Post hoc analysis performed on the main effect of Emotion revealed that Anger (0.66 µV; SE 0.27; 95% CI 0.11 to 1.20) was significantly higher than Joy (−0.45 µV; SE 0.19; 95% CI −0.84 to −0.07) (p = 0.048), whereas Sadness (1.06 µV; SE 0.60; 95% CI −0.18 to 2.30) was significantly higher than Joy (p = 0.004) and Neutral stimuli (−0.07 µV; SE 0.12; 95% CI −0.32 to 0.17) (p = 0.042). Noteworthy, post hoc comparisons conducted on the interaction Stimuli-Age by Group revealed that TG had higher Corrugator EMG activity during the viewing of Teenager-stimuli (0.34 µV; SE 0.21; 95% CI −0.09 to 0.78) than during the observation of Adult-stimuli (0.02 µV; SE 0.26; 95% CI −0.51 to 0.56) (p = 0.002) (see Figure 2, panel A).

**Figure 3:**
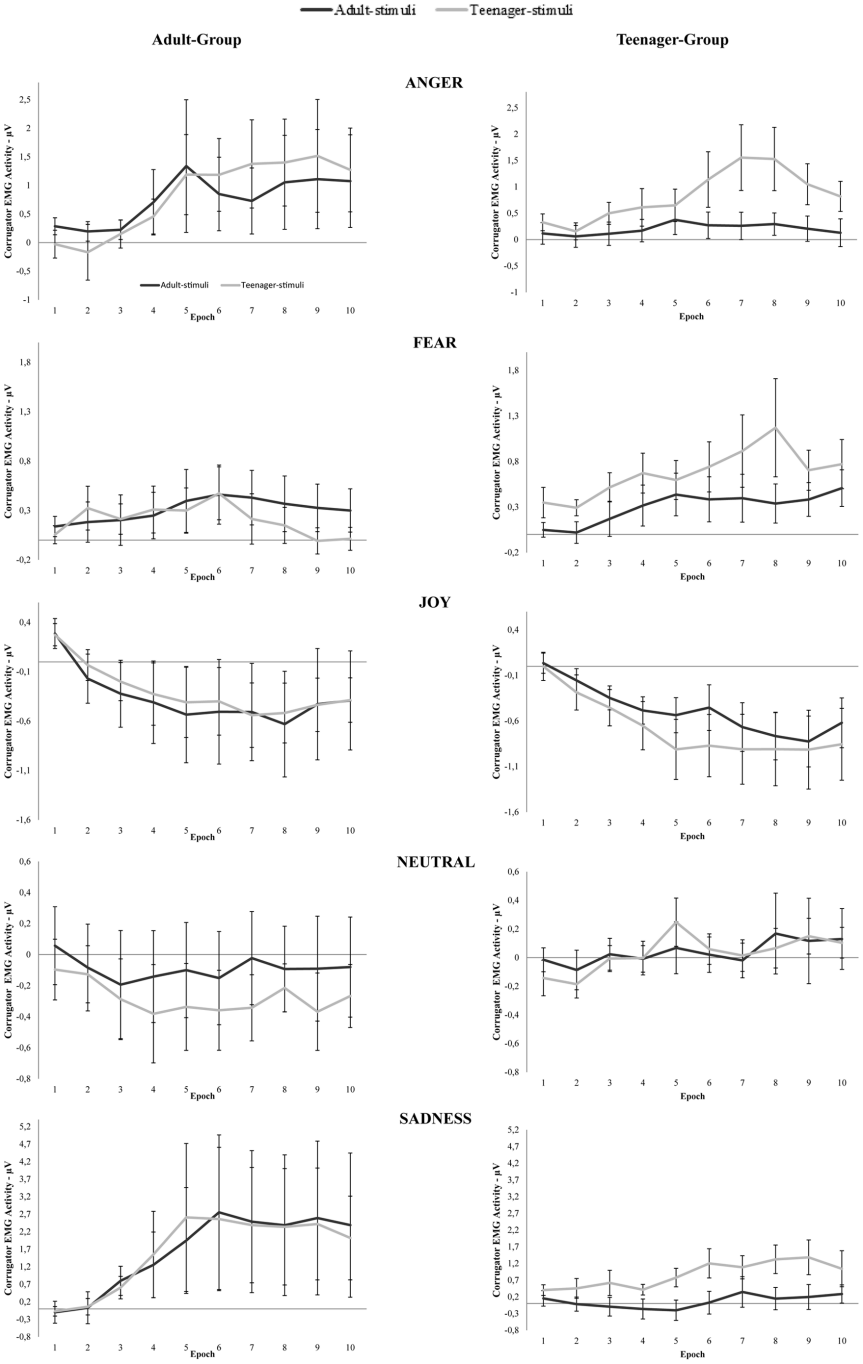
Corrugator EMG activity. Adult-Group and Teenager-Group Corrugator EMG activity in response to Adult-stimuli and Teenager-stimuli in function of time (10 epochs lasting 0.5 sec each) and emotions. Error bars represent SE.

Post hoc comparisons performed on the interaction Stimuli-Age by Epoch demonstrated that, regarding Adult-stimuli, the first 4 epochs (0–2 sec) were significantly different from the last 6 epochs (2,5–5 sec) (all p_s_<0.032). The same trend was present also among Teenager-stimuli (all p_s_<0.050). Comparing Adult-stimuli and Teenager-stimuli, the Corrugator EMG activity appeared to be significantly higher for Teenager-stimuli than Adult-stimuli from epoch 6 to epoch 9 (3–4,5 sec) (all p_s_<0.027). Post hoc analysis conducted on the interaction Emotion by Epoch revealed that, consistent with what previously described about the main effect of Emotion, Corrugator EMG activity in response to angry facial expressions was significantly higher than that recorded after exposure to joy facial expressions from epoch 5 to epoch 10 (2,5–5 sec) (all p_s_<0.049). Similarly, Corrugator EMG activity following sadness facial expressions presentation was significantly higher than that recorded in response to joy facial expressions from epoch 4 to epoch 10 (2–5 sec) (all p_s_<0.014). Moreover, Corrugator EMG activity evoked by sadness facial expressions resulted significantly higher than that recorded during neutral facial expressions observation from epoch 5 to epoch 10 (2,5–5 sec) (all p_s_<0.013). For a detailed graphical representation of Corrugator EMG activity exhibited by both experimental groups to Adult- and Teenager-stimuli in function of time and emotion see Figure 3.

Repeated measures ANOVA conducted on Zygomaticus EMG activity showed that the main factor Emotion (F_4,136_ = 8.02 p = 0.000; n^2^_p_ = 0.19) and the interaction Emotion by Epoch (F_36,1224_ = 2.94 p = 0.000; n^2^_p_ = 0.08) were significant. Post hoc comparisons conducted on the main effect of Emotion revealed that Joy (0,65 µV; SE 0.30; 95% CI 0.04 to 1.25) was significantly higher than all other stimuli (Anger: −0,36 µV; SE 0.08; 95% CI −0.54 to −0.18; Fear: −0.29 µV; SE 0.06; 95% CI −0.42 to −0.17; Neutral: −0.22 µV; SE 0.04; 95% CI −0.30 to −0.15; Sadness: −0.26 µV; SE 0.06; 95% CI −0.40 to −0.14) (all p_s_<0.001). Post hoc analysis conducted on the interaction Emotion by Epoch revealed that Zygomaticus EMG activity after joy facial expressions observation was significantly higher than that recorded in response to all other facial expressions (anger, fear, neutral and sadness) from epoch 4 to epoch 10 (2–5 sec) (all p_s_<0.002).

### RSA results

Since 2 participants were discarded (anomalous ECG was found in one participant, while outlier values ±2 SD were detected for another) ECG data from 38 participants (19 TG, 19 AG) were included in the ECG analyses. A repeated measures ANOVA was conducted with Group (TG, AG) as between-factor and Stimuli-Age (Teenager-stimuli, Adult-stimuli) and Emotion (Anger, Fear, Joy, Sadness) as within-factors. Results showed that the main factor Stimuli-Age (F_1,36_ = 5.35 p<0.027; n^2^_p_ = 0.13) as well as the interaction Group by Stimuli-Age (F_1,36_ = 11.04 p<0.002; n^2^_p_ = 0.24) were significant. Post-hoc comparison performed on Stimuli-Age main effect revealed that, regardless of group membership, all participants showed higher RSA response to Adult-stimuli [0.06 Δ ln(msec)^2^; SE 0.04; 95% CI −0.02 to 0.14] than to Teenager-stimuli [−0.05 Δ ln(msec)^2^; SE 0.04; 95% CI −0.12 to 0.02] (p<0.027). Of most interest, post-hoc analyses performed on the significant Group by Stimuli-Age interaction (see Figure 2, panel B) highlighted that AG RSA response to Adult-stimuli [0.11 Δ ln(msec)^2^; SE 0.06; 95% CI −0.002 to 0.22] was higher than to Teenagers-stimuli [−0.15 Δ ln(msec)^2^; SE 0.05; 95% CI −0.25 to −0.04] (p<0.000). Moreover, AG RSA response to Teenager-stimuli resulted to be significantly lower than that exhibited by TG in response to Adult Stimuli-Age [0.003 Δ ln(msec)^2^; SE 0.06; 95% CI −0.11 to 0.12] (p<0.026).

## Discussion

The present study investigated, for the first time, the effect of Age-group membership on both facial mimicry and autonomic regulation to emotional facial expressions. To this purpose, teenager and adult participants viewed five facial expressions (Anger, Fear, Joy, Sadness and Neutral) performed by teenager and adult models, while Facial EMG and RSA were recorded.

At first glance, results seem to point towards a role of Stimuli-Age in inducing differential physiological responses in all participants. This finding, however, could be better understood considering the significant interaction highlighted between group membership and Stimuli-Age. Specifically, TG showed higher Corrugator EMG response to Teenagers-stimuli with respect to Adult-stimuli, whereas an undifferentiated Corrugator EMG response to all stimuli was detected for AG. On the other hand, AG manifested a higher RSA-response to Adult-stimuli with respect to Teenagers-stimuli, whereas TG exhibited the same RSA-response to all stimuli. Taken together, these results demonstrated the existence of an Age-group membership effect on facial mimicry (i.e., facial EMG) as well as on autonomic regulation (i.e., RSA-response). Noteworthy, the basic physiological mechanism by means of which the Age-group membership effect emerged diverges in the two populations. Indeed, teenager participants showed stronger facial mimicry response to their peers, whereas adult participants exhibited higher RSA-response to adult facial expressions. These divergent physiological responses cohere with previous literature, documenting the existence of different neural activation patterns in young and adults during perception of emotional expressions. When looking at negative facial expressions, younger individuals, activated the right amygdala, which is a critical substrate for emotion perception and, through its connectivity to motor cortex, for facially displayed emotions [38]. On the other hand, older people exhibited, in response to the same stimuli, a higher activation of the prefrontal and the right anterior-ventral insular cortices [11], [39] which are, respectively, implicated in emotional regulation [40] and autonomic arousal [41]. The different physiological modality through which young and adults respond to peers' emotional expressions are likely to reflect two different ways to engage in social interactions with age peers. These two different modalities are not mutually exclusive but they could be recruited in a greater or lesser extent depending on the social relationship in which individuals are involved. On the one hand, teenagers, taking advantage of enhanced facial mimicry, would show greater emotional reciprocity and empathic understanding to peers' emotions [16]. Accordingly, previous studies conducted on the influence of group membership on facial mimicry, demonstrated a higher facial mimicry to in-group than to out-group members' negative facial expressions [17]–[19]. This effect was interpreted as the consequence of an unconscious physiological mechanism facilitating an immediate understanding of others' emotional state, hence promoting social cohesion and interpersonal relationship among individuals belonging to the same social group [17]–[19]. Recently, a different interpretation of Corrugator mimicry of negative facial expressions has been proposed [42]. In this study the automatic facial mimicry of negative emotions has been associated to antisocial processes. However, due to the paradigm used, possible influences related to the same social group membership were not considered. This is particularly important because, especially during adolescence, group membership plays a crucial role in social behaviours: relationships are focussed on establishing deep social relations with peers and young individuals become more autonomous and independent from parents. From childhood to adolescence, friendships evolve into more intimate, supportive and communicative relationships [43]. This relational reorientation and refinement support the emergence of novel social competences and greater susceptibility to peer influence, sustained by neural network re-organization [44], [45]. Accordingly, a wealth of data demonstrates that emotional expression recognition ability develops long into adolescence and even in early adulthood [44]. Based on these considerations and previous empirical studies focusing on group membership, we are inclined to consider that the higher Corrugator EMG activity showed by Teenager-Group to peers' facial expressions would reflect an automatic and unconscious mechanisms underlying the empathic understanding of in-group members emotions, rather than a sign of antisocial behaviours. Further studies however are warranted in order to disentangle this issue. On the other hand, adults by means of a higher RSA-response, would recruit stronger self-regulation resources in response to peers' emotions [23]. In adulthood social networks narrow, social roles change becoming more strict and relevant, the investment in meaningful relationships increases and interactions with unfamiliar peers become more structured and hierarchically organized [46]. In this scenario, self-regulation responses are crucially involved during social relations with adult peers.

Some considerations about limitations and improvements concerning the described results have nevertheless to be done. First, the Age-group membership effect on Corrugator activity turned out to be generalized to all facial expressions and not limited to the negative ones as reported in earlier studies [18], [19], [47]. However, it is clear from our results that in Teenager-Group (in which an Age-group membership effect for Corrugator EMG activity was detected), the greater muscular activation in response to teenagers' facial expressions was present only for the expected angry, fear and sadness emotions. It could be possible that the imbalanced number of negative (Anger, Fear, Sadness), positive (Joy) and neutral facial expressions employed in the study as well as the absence of an Age-group membership effect among adults' Corrugator EMG activation prevented the emergence of an Age-group membership effect specific for negative emotions. Furthermore, results obtained for Zygomaticus EMG activity enhance the relevance of the Age-group membership influence on facial mimicry of negative expressions. According to recent evidence about the relation between mimicry of smiles and age [48], an effect of group membership on Zygomatic muscle (whose greater activity was exclusively related with Joy facial expressions) was not found. This result confirmed that, thanks to their positive and powerful affiliative nature, joy facial expressions would overrule Age-group boundaries. A further limitation of the present study was the narrow number of muscles recorded. The inclusion of other muscles involved in negative and positive facial expressions (e.g., *Medial frontalis, Depressor anguli* and *Orbiculari Oculi* muscles) could effectively extend and clarify the role of Age-group membership in EMG activations to negative and positive facial expressions. Finally, due to a high number of possible interfering social conditions, it was not possible to control participants' actual levels of interaction with people belonging to the same and/or other age-group. In this experimental protocol we did not investigate the effect of Gender-group membership, rather we balanced participants and stimuli for gender in order to avoid its potential confounding effect on physiological responses. As line for future research, further studies should investigate possible interactions between Age-group and Gender-group membership on facial mimicry and autonomic responses.

In conclusion, our results demonstrate that Age-group membership influences not only the explicit recognition and attention processes paid to emotional facial expressions [11], but also the implicit physiological responses to them, which induce different modalities of socio-emotional interpersonal interactions with age peers.

## Supporting Information

Movie S1 **Illustrative Teenager-stimulus.**
(AVI)

Movie S2 **Illustrative Adult-stimulus.**
(AVI)

Text S1 **Stimuli construction and validation.** Detailed description of procedures followed for stimuli construction and validation.
(DOCX)

Text S2 **RSA Suppression.** Detailed description of RSA suppression analyses conducted on Baseline RSA values.
(DOCX)
