# Supplementary material for: When Age Matters: Differences in Facial Mimicry and Autonomic Responses to Peers' Emotions in Teenagers and Adults
Source: PLoS One. 2014 Oct 22;9(10):e110763. doi: 10.1371/journal.pone.0110763 (PMC4206508; doi:10.1371/journal.pone.0110763)
Supplement: Text S1 — Stimuli construction and validation. Detailed description of procedures followed for stimuli construction and validation. (DOCX) [file pone.0110763.s003.docx]

**Supplementary Information S1**

**Stimuli construction and validation**

All models involved in stimuli construction and validation were unfamiliar to participants who took part to the experimental procedure described in the main text. Written informed consent was collected from all volunteers, or their parents in case of underage individuals, involved in stimuli construction and validation.

Coloured pictures of teenagers (n = 15; 7 males; 15-19 years old; mean age 16.87 years SE 0.40) and adults’ (n = 17; 9 males; 50-55 years old; mean age 52.50 SE 0.63) faces were taken with a digital camera. Models were asked to express 4 emotions (anger, fear, joy, sadness) and two versions of neutral facial expressions. Photographs were elaborated with Adobe Photoshop to create a gray background and to uniform size (1000×666 pixels), brightness and contrast. 317 Morphed videos were then constructed using a face-morphing software (Squirlz Morph, <http://www.xiberpix.net/SqirlzMorph.html>).

Sixty-eight volunteers, homogeneously assigned to a specific age range from 15 years to 55 years (15-19; 20-24; 25-29; 30-34; 35-39; 40-44; 45-49; 50-55 years), took part to stimuli validation. The first and the last two age ranges matched the age of the Teenager-Group and the Adult-Group involving in the experimental study described in the main manuscript.

Volunteers were instructed to look carefully at each video and to press the spacebar as soon as they recognized the expressed emotion (Reaction Time, RT) choosing among six alternative labels displayed on the screen [“anger”, “fear”, “joy”, “sadness”, “neutral” and “?” (i.e., “I don’t know”)] (Accuracy). Furthermore, volunteers had to state if the model presented in the stimuli was or not a peer (i.e., someone of same age).

The exact age matching between participants engaged in the experiment (Teenager-Group 15-19 years; Adults-Group 45-55 years) and the models of the stimuli (Teenager-stimuli: 15-19 years of the model; Adult-stimuli: 45-55 years of the model) was a core element of our experimental hypothesis. For this reason, we firstly selected only the morphed videos performed by models considered as a peer by the majority (at least 60%) of the volunteers belonging to the age ranges of interest (15-19 and 45-55). Following these criteria 147 videos were retained. At a second stage, 12 morphed videos (6 modelled by teenagers: Teenager-stimuli; 6 modelled by adults: Adult-stimuli) were selected for each expression (Joy, Fear, Sadness, Anger, Neutral), counterbalancing them for accuracy rate and reaction time, resulting in a final number of 60 validated stimuli. To ensure that the final selected stimuli were homogeneously recognized and did not differ for intensity, we performed five independent t-tests on Accuracy Rate and RTs contrasting Teenager-stimuli and Adult-stimuli. We found no significant differences between Teenager-stimuli and Adult-stimuli (all p_s_ > 0.05) in each emotion condition (see Table S1a and Table S1b).

|  | **Mean Teenager-stimuli (SE)** | **Mean Adult-stimuli (SE)** | **t** | **gl** | **p** |
| --- | --- | --- | --- | --- | --- |
| **Anger** | 82.11 % (4.35) | 81.98 % (4.30) | 0.02 | 10 | 0.98 |
| **Fear** | 88.97 % (3.81) | 86.52 % (7.18) | 0.30 | 10 | 0.77 |
| **Joy** | 100 % (0) | 100 % (0) | -- | 10 | -- |
| **Neutral** | 91.18 % (2.21) | 93.87 % (0.96) | 1.12 | 10 | 0.29 |
| **Sadness** | 88.47 % (5.88) | 88.91 % (3.06) | 0.67 | 10 | 0.95 |

**Table S1a:** Table shows mean Accuracy rate for Teenager-stimuli and Adult-stimuli. Results of independent sample t-tests comparing Accuracy rate for Teenager-stimuli and Adult-stimuli are reported.

|  | **Mean Teenager-stimuli (SE)** | **Mean Adult-stimuli (SE)** | **t** | **gl** | **p** |
| --- | --- | --- | --- | --- | --- |
| **Anger** | 2.90 sec (0.10) | 2.86 sec (0.07) | 0.31 | 10 | 0.76 |
| **Fear** | 2.66 sec (0.22) | 2.70 sec (0.13) | - 0.15 | 10 | 0.88 |
| **Joy** | 1.64 sec (0.03) | 1.78 sec (0.09) | - 1.47 | 10 | 0.17 |
| **Neutral** | 3.02 sec (0.07) | 2.90 sec (0.06) | 1.31 | 10 | 0.22 |
| **Sadness** | 2.55 sec (0.13) | 2.74 sec (0.09) | - 1.17 | 10 | 0.27 |

**Table S1b:** Table show mean Reaction Times for Teenager-stimuli and Adult-stimuli. Results of independent sample t-tests comparing Reaction Times for Teenager-stimuli and Adult-stimuli are reported.
