# Supplementary material for: When Age Matters: Differences in Facial Mimicry and Autonomic Responses to Peers' Emotions in Teenagers and Adults
Source: PLoS One. 2014 Oct 22;9(10):e110763. doi: 10.1371/journal.pone.0110763 (PMC4206508; doi:10.1371/journal.pone.0110763)
Supplement: Text S2 — RSA Suppression. Detailed description of RSA suppression analyses conducted on Baseline RSA values. (DOCX) [file pone.0110763.s004.docx]

**Supporting Information S2**

**RSA suppression**

Previous evidence showed that RSA amplitude is significantly reduced during tasks requiring sustained attention [1,2]. In order to detect a possible RSA suppression in our experiment, we compared RSA values of Baseline1 and Baseline2. Repeated measures ANOVA was conducted on Baseline1 and Baseline2 RSA values, with Group (TG, AG) as between-factor and Baseline (1, 2) as within-factor. Results showed a significant main effect of Group (F_1,36_= 45.78 p < 0.0001; ƞ^2^_p_ = 0.56) and Baseline (F_1,36_= 10.77 p < 0.01; ƞ^2^_p_ = 0.23). Post-hoc comparisons revealed that TG had a higher RSA value with respect to AG [TG: 6.37 ln(msec)^2^; SE 0.20; 95% CI 5.96 to 6.78; AG: 4.44 ln(msec)^2^; SE 0.20; 95% CI 4.03 to 4.85; p<0.0001]. Moreover, post-hoc conducted on the main effect of Baseline highlighted that RSA value of Baseline1 was higher than that of Baseline2 in all participants [Baseline1: 5.52 ln(msec)^2^; SE 0.15; 95% CI 5.22 to 5.83; Baseline 2: 5.29 ln(msec)^2^; SE 0.14; 95% CI 5.00 to 5.58; p < 0.05). To better explore the occurrence of RSA suppression throughout the experiment, we performed the same analysis comparing the first and the last Age-blocks. Repeated measures ANOVA was performed with Group (TG, AG) as between-factor and Age-block (1^st^ Block, 8^th^ Block) as within-factor. Results showed, again, a significant main effect of Group (F_1,36_= 42.61 p < 0.0001; ƞ^2^_p_ = 0.54) and Age-block factors (F_1,36_= 5.73 p < 0.05; ƞ^2^_p_ = 0.14). Post-hoc comparisons confirmed a higher RSA value of the TG [TG: 6.23 ln (msec)^2^; SE 0.20; 95% CI 5.82 to 6.63; AG: 4.38 ln (msec)^2^; SE 0.20; 95% CI 3.98 to 4.79; p < 0.0001]. Furthermore, RSA value in the first Age-block was higher than that in the last Age-block [1^st^ Block: 5.37 ln (msec)^2^; SE 0.14; 95% CI 5.10 to 5.65; 8^th^ Block: 5.24 ln (msec)^2^; SE 0.15; 95% CI 4.93 to 5.54; p < 0.05].

These analyses revealed two main findings. First, we confirmed the well known decline of RSA amplitude with age [3,4]. Adults’ RSA values, recorded both in rest conditions (Baseline1 and Baseline2) and in emotional conditions (Age-blocks), were lower than teenagers’ ones. Second, a suppression effect was detected in all participants throughout the experiment. RSA values at the beginning of the experiment (Baseline1 and 1^st^ Block) were higher than those recorded at its end (Baseline2 and 8^st^ Block). An effect of sustained attention is coherent with our experimental task, in which participants were instructed to carefully look at the videos. In order to avoid possibly confounding effects originating from age difference and suppression effect on RSA values, we performed a proper normalization procedure (see the main text for a detailed description).

**References**

1. Suess PE, Porges SW, Plude DJ (1994) Cardiac vagal tone and sustained attention in school-age children. Psychophysiology 31: 17–22. Available: http://www.ncbi.nlm.nih.gov/pubmed/8146250. Accessed 30 July 2013.

2. Porges SW (1995) Orienting in a defensive world: Mammalian modifications of our evolutionary heritage. A Polyvagal Theory. Psychophysiology 32: 301–318. Available: http://doi.wiley.com/10.1111/j.1469-8986.1995.tb01213.x. Accessed 14 November 2012.

3. De Meersman RE, Stein PK (2007) Vagal modulation and aging. Biol Psychol 74: 165–173. Available: http://www.ncbi.nlm.nih.gov/pubmed/17045727. Accessed 20 August 2013.

4. Dywan J, Mathewson KJ, Choma BL, Rosenfeld B, Segalowitz SJ (2008) Autonomic and electrophysiological correlates of emotional intensity in older and younger adults. Psychophysiology 45: 389–397. Available: http://www.ncbi.nlm.nih.gov/pubmed/18221446. Accessed 17 August 2013.
